# Surgical outcomes and perioperative risk factors of patients with interstitial lung disease after pulmonary resection

**DOI:** 10.1186/s13019-024-02508-1

**Published:** 2024-02-08

**Authors:** Chuan Huang, Yao-Guang Sun, Chao Ma, Peng Jiao, Qing-Jun Wu, Wen-Xin Tian, Han-Bo Yu, Hong-Feng Tong

**Affiliations:** Department of Thoracic Surgery, Beijing Hospital, National Center of Gerontology, Institute of Geriatric Medicine, Chinese Academy of Medical Sciences, NO.1 Da Hua Road, Dongdan, Beijing, 100730 P.R. China

**Keywords:** Interstitial lung disease, Pulmonary resection, Complication, Acute exacerbation

## Abstract

**Background:**

Patients of interstitial lung disease (ILD) combined with pulmonary lesions are increasingly common in clinical practice. Patients with ILD are at significantly higher risk for complications after pulmonary resection (including lobectomy and sublobar resection), especially acute exacerbations of ILD (AE-ILD). The purpose of this study is to summarize the short-term and long-term outcomes after pulmonary resection in ILD patients and to analyze the clinical factors affecting surgical safety.

**Methods:**

From January 2004 to January 2022, a total of 78 patients who were diagnosed with ILD and underwent pulmonary resection at our center were enrolled in this study. Clinical data, pathological findings, surgical procedures, and intraoperative safety of these patients were collected retrospectively. Postoperative 90-day complications and mortality, long-term surgical outcomes from postoperative 90 days to 24 months, and changes in ILD condition were investigated. Logistic regression analysis was used to identify the risk factors associated with postoperative complications.

**Results:**

The median age of patients was 66.5 (range 33–86) years, 82.1% (64/78) of patients were male, and 78.2% (61/78) of patients had comorbidities. Idiopathic ILD and secondary ILD accounted for 86% and 14%, thoracotomy and video-assisted thoracoscopic surgery accounted for 12.8% and 87.2%, and lobectomy and sublobar resection accounted for 37.2% and 62.8%, respectively. Postoperative 90-day complications occurred in 25.6% (20/78) of patients, with pulmonary complications and AE-ILD occurring in 15.4% and 9.0% of patients, respectively. The postoperative 90-day mortality rate was 5.1% (4/78), and the cause of death was AE-ILD. Exacerbation of ILD or other complications occurred in 12.8% (10/78) of patients from postoperative 90 days to 24 months. Univariate logistic regression analysis showed that comorbidity, extent of resection, systemic lymph node dissection, operation time, intraoperative blood loss, and pathology of pulmonary lesion were associated with postoperative 90-day complications. In multivariate logistic regression analysis, age-adjusted Charlson Comorbidity Index and intraoperative blood loss were identified as independent risk factors of postoperative 90-day complications.

**Conclusions:**

Patients with ILD have a significantly higher risk of postoperative 90-day complications and mortality after pulmonary resection, especially pulmonary complications and AE-ILD. After postoperative 90 days, the risk of deterioration of pulmonary status remains high, including exacerbation of ILD and complications associated with long-term use of glucocorticoids and immunosuppressant. Age, comorbidity and intraoperative blood loss are high risk factors for postoperative 90-day complications.

## Introduction

Interstitial lung disease (ILD) is a heterogeneous group of diffuse lung diseases that mainly involve the pulmonary interstitium, causing loss of alveolar-capillary functional units and often leading to restrictive ventilatory dysfunction and diffusion impairment [1]. The etiology and classification of ILD are numerous and complex. Idiopathic ILD accounts for approximately 60% of cases and its etiology is unclear. Common types include idiopathic pulmonary fibrosis (IPF), idiopathic nonspecific interstitial pneumonia (iNSIP), desquamative interstitial pneumonia (DIP), etc [2, 3]. Secondary ILD accounts for about 40% of cases and its causes include occupational or environmental factors, medication, viral or bacterial infection, connective tissue disease, etc. Connective tissue diseases related interstitial lung disease (CTD-ILD) is a common type, and is often induced by systemic sclerosis, rheumatoid arthritis, polymyositis and dermatomyositis, and Sjogren syndrome [4, 5].

The risk of lung cancer (LC) was significantly increased in ILD patients, and the incidence of ILD combined with LC was up to 10-20%, among which the incidence of lung cancer in IPF patients and CTD-ILD patients was 13-20% and 5%, respectively [6]. During the treatment of lung cancer, drugs, chest radiotherapy, and thoracic surgery may induce the worsening of interstitial pneumonia, of which pulmonary resection is an important high-risk factor. Acute exacerbation of ILD (AE-ILD) may occur not only after major pulmonary surgery such as radical resection of primary tumor plus systemic lymph node dissection, but also after minor pulmonary surgery such as bronchoscopy and lung biopsy [7]. The incidence and mortality of AE-ILD after pulmonary resection may be as high as 16% and 60%, respectively [8].

Although there have been many studies on the perioperative safety of ILD patients, high-quality clinical data are still lacking in several fields. Other than IPF, postoperative complications and acute exacerbations of other ILD types (e.g., iNSIP and CTD-ILD) have been rarely reported. Most studies have focused on the short-term postoperative outcomes, such as postoperative 90-day morbidity and mortality, while few studies focused on the long-term outcomes, such as complications beyond postoperative 90-day, subsequent changes in condition and treatment of ILD, and effects of ILD on tumor prognosis [9, 10]. The purpose of this study is to summarize the short-term and long-term outcomes after pulmonary resection in ILD patients and to analyze the clinical factors affecting surgical safety, in order to provide evidence for the surgical treatment of ILD patients.

## Materials and methods

### Patients’ selection and diagnostic criteria

Between January 2004 and January 2022, a total of 4979 pulmonary resections were performed in the Department of Thoracic Surgery at Beijing Hospital, of which 78 patients with a confirmed diagnosis of ILD were consecutively selected. Clinical characteristics, imaging and pathological features, surgical procedures, postoperative morbidity and mortality, subsequent treatment and outcomes of these patients were retrospectively reviewed. All ILD was diagnosed by high-resolution computed tomography (HRCT). The diagnostic criteria for idiopathic ILD adopted the international multidisciplinary classification of the idiopathic interstitial pneumonias recommended by the American Thoracic Society/European Respiratory Society in 2013 [3]. The diagnosis of secondary ILD was based on in a multidisciplinary collaboration, usually including physicians in the departments of respiratory, rheumatology, thoracic surgery, radiology, pathology, etc., and lung biopsy was performed when necessary to identify the etiology of ILD. For pathologically confirmed lung cancer, tumor stage was determined according to 8th edition of American Joint Committee on Cancer /Union for International Cancer Control TNM (Tumor, Node, Metastasis) staging system [11].

### Preoperative evaluation

Preoperative chest CT, abdominal ultrasound, cranial magnetic resonance image (MRI) or CT, and whole-body bone scan were performed to determine the nature of pulmonary lesion, evaluate tumor stage and exclude metastasis. Positron emission tomography was performed when necessary. Arterial blood gas, pulmonary function and echocardiography were performed to evaluate surgical tolerance. Patients with critical pulmonary function impairment underwent cardiopulmonary exercise test to determine whether they could tolerate pulmonary resection. Patients’ comorbidities were evaluated and treated preoperatively, and the age-adjusted Charlson Comorbidity Index was used for preoperative comorbidities evaluation [12]. The preoperative unstable or aggravated of ILD was defined as an exacerbation within three months prior to surgery, such as aggravation of respiratory symptoms, worsening of pulmonary radiologic manifestations, increase in the dose or type of medication, occurrence of pulmonary infection or exacerbation of other pulmonary comorbidities, decline in pulmonary function test, or occurrence of AE-ILD. The diagnosis of unstable or aggravated ILD was determined after discussion by the multidisciplinary team at our center.

### Surgical techniques and perioperative management

Anesthesia was combined intravenous and inhalation general anesthesia with double-lumen endotracheal intubation or single-lumen tube using bronchial blocker to maintain single-lung ventilation. All patients routinely underwent video-assisted thoracoscopic surgery (VATS) using single-utility port technique. As for the surgical approach, one 1-centimeter (cm) incision in the 7th or 8th intercostal space along the midaxillary line as the observation port and one 2–3 cm incision in the 3rd to 5th intercostal space along the anterior axillary line as the operation port. Depending on location, size, and pathology of the pulmonary lesions, preoperative lung function and comorbidities, lobectomy, segmentectomy or wedge resection were performed. Systemic lymph node dissection was performed if pulmonary lesion was diagnosed as malignant by intraoperative rapid frozen pathology. For right-sided lung cancer, groups 2, 4, 7, 8, 9, 10 and intrapulmonary lymph nodes were dissected. For left-sided lung cancer, groups 4, 5, 6, 7, 8, 9, 10 and intrapulmonary lymph nodes were dissected. At the end of the operation, one 28Fr drainage tube was placed in pleural cavity. After the operation, the patients were routinely extubated and returned to the thoracic surgery ward. Treatment such as prophylaxis of surgical site infection, nebulized inhalation, and assistance in expectoration were routinely given postoperatively. The patient received a chest radiograph on postoperative day 1–2, and if there is no active bleeding or air leakage, the pleural drain can be removed on postoperative day 2–3.

### Evaluation of postoperative complications

Postoperative complications and mortality were recorded based on the definitions recommended by General Thoracic Surgery Database of the Society of Thoracic Surgeons [13]. AE-ILD was diagnosed using the definition and diagnostic criteria recommended by International Multidisciplinary Working Group in 2016 [14]. Postoperative complications were divided into short-term complications (within postoperative 90-day) and long-term complications (beyond postoperative 90-day). The clavien-dindo classification of surgical complications was used to grade the severity of postoperative complications [15]. The diagnosis of ILD and AE-ILD was determined after discussion by the multidisciplinary team at our center.

### Follow-up

Follow-up was conducted through regular outpatient visits, telephone calls or letters to record postoperative outcomes and oncological efficacy. All patients were requested to visit the respiratory clinic regularly to follow up changes in clinical symptoms and chest imaging of ILD and to adjust its treatment. For patients with pathologically confirmed malignant tumors, Chest CT, ultrasound of supraclavicular lymph nodes and abdominal organs, and blood tumor markers were reviewed every 3 months in the first two years, every 6 months in the next three years, and then every 1 year after 5 years postoperatively. Cranial MRI and whole-body bone scan were reviewed annually or when there were corresponding symptoms. The last follow-up date was January 31, 2023.

### Statistical analysis

Categorical variables were presented as numbers and percentages. The continuous variables were tested for normality and variance homogeneity. The continuous variables of normal distribution and non-normal distribution are expressed as mean values ± standard deviation, median and range, respectively. The Pearson’s χ2 test or fisher exact test was applied to compare the distribution of categorical variables between the group with postoperative 90-day complications and the group without postoperative 90-day complications. The continuous variables that obeyed normal distribution and homogeneity of variance were compared by *t*-test, while continuous variables that did not obey normal distribution and homogeneity of variance were compared by Mann-Whitney U test. Odds ratios (OR) and 95% confidence intervals (CI) were calculated by unconditional logistic regression to evaluate risks of complication adjusting for potential confounders (e.g., age.). Stepwise backward multiple logistic regression analysis was used to identify risk factors that were independently associated postoperative 90-day complications. All *p* values were two-sided and less than 0.05 was considered statistically significant. All statistical analyses were performed using the Statistical Package for the Social Sciences (SPSS 25.0, IBM).

## Results

### Clinical characteristics

A total of 78 patients who were diagnosed with ILD and underwent pulmonary resection were enrolled in this study. The patients’ median age was 66.5 (range, 33–86) years, and 82.1% (64/78) of the patients were male. 56% (44/78) of the patients had smoking history and median smoking index was 40 (range, 10–150) package-years. 78% (61/78) of the patients had comorbidities, including hypertension (*n* = 32), coronary heart disease (*n* = 20), diabetes mellitus (*n* = 23), chronic obstructive pulmonary disease (*n* = 14), autoimmune disease (*n* = 10), and other malignancy (*n* = 3). The age-adjusted Charlson Comorbidity scores of the patients ranged from 0 to 7 and included 7 cases of 0, 7 cases of 1, 11 cases of 2, 19 cases of 3, 12 cases of 4, 14 cases of 5, 7 cases of 6 and 1 case of 7. Ten patients (12.8%) had a history of long-term exposure to respiratory pollutants. 82% (64/78) of patients had ventilation dysfunction, 68% (53/78) of patients had small airway dysfunction and 71% (55/78) of patients had diffusion dysfunction.

82% (64/78) of the patients were diagnosed with idiopathic ILD of unknown etiology, including 40 cases of IPF, 23 cases of iNSIP, and 1 case of desquamative interstitial pneumonia. Three patients were diagnosed with hypersensitivity pneumonitis. 14% (11/78) of the patients were diagnosed with CTD-ILD, including systemic sclerosis in 2 cases, rheumatoid arthritis in 2 cases, Sjogren syndrome in 2 cases, systemic lupus erythematosus in 1 case, anti-neutrophil cytoplasmic antibodies associated vasculitis in 1 case, immunoglobulin G4 -related lung disease in 1 case, and 2 cases of unknown cause. 35% (27/78) of the patients had long-term use of glucocorticoids or immunosuppressants, and 23% (18/78) of the patients had long-term use of antifibrotic drugs.

64% (50/78) of the patients were pathologically diagnosed with lung cancer, including adenocarcinoma (42%, 21/50), squamous cell carcinoma (32%, 16/50), small cell carcinoma (16%, 8/50), small cell carcinoma mixed squamous cell carcinoma (2%, 1/50), large cell neuroendocrine carcinoma (4%, 2/50), mucinous adenocarcinoma (2%, 1/50), and mucoepidermoid carcinoma (2%, 1/50). The pathological stages included 19 cases (38%) of stage IA, 10 cases (20%) of stage IB, 6 cases (12%) of stage IIB, 9 cases (18%) of stage IIIA, 1 case (2%) of stage IIIB, and 5 cases (10%) of stage IV. EGFR mutations were detected in 12% (6/50) of patients. Postoperatively, 14 patients received adjuvant chemotherapy, 1 patient received epidermal growth factor receptor tyrosine kinase inhibitor therapy, and 2 patients received adjuvant radiotherapy.

10% (8/78) of the patients were pathologically diagnosed with benign lesions, including inflammatory pseudotumor (*n* = 3), tuberculosis (*n* = 2), sclerosing hemangioma (*n* = 1), hamartoma (*n* = 1), and sarcoidosis (*n* = 1). 26% (20/78) of patients did not have primary pulmonary lesion, and wedge resections were performed to determine the pathology of ILD. The clinical characteristics and pathological results of the patients were shown in Table 1.

**Table 1:** Clinical characteristics of ILD patients who have undergone pulmonary resection

	Whole group *N* = 78 (%)	Non-POC group *N* = 58 (%)	POC group *N* = 20 (%)	*P* value^a^
**Gender**				
Male	64 (82.1)	47 (81)	17 (85)	0.690
Female	14 (17.9)	11 (19)	3 (15)	
**Age(year), Median (range)**	66.5 (33–86)	66 (33–80)	73 (57–86)	**0.001** ^b^
**Smoking history**				
Current smoker	24 (30.8)	20 (34.5)	4 (20)	0.196
Ex-smoker	20 (25.6)	12 (20.7)	8 (40)	
Non-smoker	34 (43.6)	26 (44.8)	8 (40)	
Smoking index of smokers (package-years), Median (range)	40 (10–150)	40 (10–120)	50 (20–150)	0.507^b^
**Comorbidity**				
COPD	14 (17.9)	7 (12.1)	7 (35)	**0.049**
Coronary heart disease	20 (25.6)	12 (20.7)	8 (40)	0.088
Autoimmune disease	10 (12.8)	8 (13.8)	2 (10)	0.960
**Environmental exposures**	10 (12.8)	9 (15.5)	1 (5)	0.409
**Type of ILD**				
IPF	40 (51.3)	26 (44.8)	14 (70)	0.183
iNSIP	23 (29.5)	20 (34.5)	3 (15)	
CTD-ILD	11 (14.1)	8 (13.8)	3 (15)	
Other types	4 (5.1)	4 (6.9)	0 (0)	
**Scope of ILD on CT imaging**				
Localized distribution	61 (78.2)	45 (77.6)	16 (80)	1.000
Diffuse distribution	17 (21.8)	13 (22.4)	4 (20)	
**Preoperative condition of ILD**				
Stable	64 (82.1)	49 (84.5)	15 (75)	0.539
Unstable or aggravated	14 (17.9)	9 (15.5)	5 (25)	
**Preoperative PFT, Mean±SD (range)**				
FEV_1_ (% pred)	77.98±16.28	78.43±16.29	76.76±16.61	0.698
FVC (% pred)	79.32±18.01	78.34±18.65	81.97±16.31	0.446
FEV_1_/FVC (% pred)	76.21±11.04	77.71±10.46	72.16±11.82	0.054
MVV (% pred)	72.09±16.96	70.45±15.33	76.46±20.47	0.179
DLCO (% pred)	66.22±17.99	67.46±19.03	62.88±14.72	0.334
**Preoperative ABG, Mean±SD**				
PaO_2_ (mmHg)	78.95±8.99	78.45±9.37	80.42±7.79	0.412
PaCO_2_ (mmHg)	38.72±3.42	39.02±3.34	37.84±3.61	0.198
SaO_2_ (%)	95.66±1.95	95.62±1.99	95.79±1.84	0.745
**Location of Primary lung cancer**				
Right upper lobe	16 (32)	8 (24.2)	8 (47.1)	0.292^c^
Right middle lobe	4 (8)	4 (12.1)	0 (0)	
Right lower lobe	9 (18)	5 (15.2)	4 (23.5)	
Left upper lobe	11 (22)	8 (24.2)	3 (17.6)	
Left lower lobe	10 (20)	8 (24.2)	2 (11.8)	
**Pathology of pulmonary lesion**				
Benign lesion or ILD	28 (35.9)	25 (43.1)	3 (15)	**0.024**
Primary lung cancer	50 (64.1)	33 (56.9)	17 (85)	
Adenocarcinoma	21 (42)	15 (45.5)	6 (35.3)	0.405
Squamous carcinoma	16 (32)	10 (30.3)	6 (35.3)	
Large cell neuroendocrine carcinoma	2 (4)	2 (6.1)	0 (0)	
Small cell carcinoma	8 (16)	3 (9.1)	5 (29.4)	
Small cell carcinoma mixed squamous cell carcinoma	1 (2)	1 (3.0)	0 (0)	
Other types	2 (4)	2 (6.1)	0 (0)	
**Pathological TNM stage**				
I	29 (58)	18 (54.5)	11 (64.7)	0.842^c^
II	6 (12)	5 (15.2)	1 (5.9)	
III	10 (20)	7 (21.2)	3 (17.6)	
IV	5 (10)	3 (9.1)	2 (11.8)	

Statistical method: a: Pearson’s χ2 test; b: Mann-Whitney U test; c: Fisher exact test;

Abbreviation: ABG: arterial blood gas; COPD: chronic obstructive pulmonary disease; CTD-ILD: connective tissue diseases related interstitial lung disease; DLCO: diffusing capacity of carbon monoxide; FEV1: forced expiratory volume in 1 s; FVC: forced vital capacity; iNSIP: idiopathic nonspecific interstitial pneumonia; ILD: interstitial lung disease; IPF: idiopathic pulmonary fibrosis; MVV: maximal voluntary ventilation; PaO_2_: arterial oxygen pressure; PaCO_2_: arterial carbon dioxide pressure; PFT: pulmonary function test; POC: postoperative 90-day complications; SaO_2_: arterial oxygen saturation; SD: standard deviation;

### Surgical outcomes and intraoperative safety

The surgical methods of 28 patients diagnosed with benign pulmonary lesions included 1 lobectomy, 1 segmentectomy, and 26 wedge resections. The surgical methods of 50 patients diagnosed with lung cancer included 1 bilobectomy, 1 bronchial sleeve resection, 24 lobectomies, 2 lobectomies combined with segmentectomy or wedge resection, 8 segmentectomies and 14 wedge resections. In addition, one patient underwent lobectomy of left lower lobe via thoracotomy and then wedge resection of right lower lobe via VATS four months later. Surgical approaches included VATS (87.2%, 68/78) and thoracotomy (12.8%, 10/78).

The median operation time was 107.5 min (range, 15 to 440 min), median intraoperative blood loss was 50 ml (range, 5 to 700 ml), and intraoperative blood transfusion was required in 4 patients. Intraoperative thoracic adhesion was found in 9 patients and conversion to thoracotomy was required in 1 patient. The median thoracic drainage duration was 3 days (range, 1 to 13 days), median pleural drainage was 620 ml (range, 30 to 5100 ml), and median postoperative hospital stay was 10 days (range, 3 to 90 days).

### Short-term postoperative complications

Postoperative 90-day complications occurred in 25.6% (20/78) of patients, including 12 pulmonary complications, 11 cardiovascular complications, and 2 other complications. The incidence of pulmonary complications was 15.4%, including 4 cases of pulmonary air leaks, 3 cases of pleural effusion, 5 cases of pulmonary infection and 10 cases of respiratory failure. The incidence of cardiovascular complications was 14.1%, including 5 cases of sinus tachycardia, 6 cases of atrial fibrillation, 1 case of atrial bigeminy and 2 cases of heart failure. Other complications included 1 case of pleural hemorrhage and 1 case of chylothorax. According to the clavien-dindo classification of surgical complications, 20 patients with postoperative 90-day complications were classified as grade II in 9 cases (11.5%), grade III-a in 1 case (1.3%), grade IV-a in 6 cases (7.7%), grade IV-b in 1 case (1.3%), and grade V in 3 cases (3.8%). For the 40 patients with IPF, 23 patients with iNSIP, and 11 patients with CTD-ILD, the incidence of postoperative 90-day overall complications and pulmonary complications was 35% (14/40) and 20% (8/40), 13% (3/23) and 4% (1/23), 27% (3/11), and 27% (3/11), respectively. The surgical data and perioperative outcomes of the patients were shown in Table 2.

**Table 2:** Surgical data and perioperative outcomes after pulmonary resection in ILD patients

	Whole group *N* = 78 (%)	Non-POC group *N* = 58 (%)	POC group *N* = 20 (%)	*P* value^a^
**Surgical approach**				
Thoracotomy	10 (12.8)	7 (12.1)	3 (15)	1.000
VATS	68 (87.2)	51 (87.9)	17 (85)	
**Extent of resection**				
Sublobar resection^1^	49 (62.8)	41 (70.7)	8 (40)	**0.037**
Lobectomy	26 (33.3)	15 (25.9)	11 (55)	
Extended resection^2^	3 (3.8)	2 (3.4)	1 (5)	
**Systematic mediastinal lymph node dissection**	50 (64.1)	33 (56.9)	17 (85)	**0.024**
**Operation time (minute), Median (range)**	107.5 (15–440)	102.5 (15–300)	130 (60–440)	**0.012** ^b^
**Intraoperative blood loss(ml), Median (range)**	50 (5-700)	30 (5-700)	100 (20–600)	**0.000** ^b^
**Intraoperative accident or blood transfusion**	12 (15.4)	7 (12.1)	5 (25)	0.167
**Thoracic drainage volume(ml), Median (range)**	620 (30-5100)	500 (30-3000)	1025 (300–5100)	**0.001**
**Thoracic drainage duration(day), Median (range)**	3 (1–13)	3 (1–9)	4 (1–13)	**0.003**
**Postoperative hospital stay (day), Median (range)**	10 (3–90)	9 (3–90)	13 (5–41)	**0.007**
**Postoperative 30-day mortality**	3 (3.8)	0 (0)	3 (15)	**0.020**
**Postoperative 90-day mortality**	3 (3.8)	0 (0)	3 (15)	**0.020**

Note: 1: Sublobar resection includes wedge resection and segmentectomy; 2: extended resection includes bronchial sleeve resection, lobectomy combined with segmentectomy or wedge resection, and bilobectomy;

Statistical method: a: Pearson’s χ2 test; b: Mann-Whitney U test;

Abbreviation: AE-ILD: acute exacerbation of interstitial lung disease; SD: standard deviation; VATS: video-assisted thoracoscopic surgery;

The postoperative 90-day mortality rate was 5.1% (4/78), and the cause of death was AE-ILD. A total of 7 patients developed AE-ILD, including 5 cases of IPF and 2 cases of CTD-ILD, and the median time to onset of AE-ILD was 3 days (range 2–52 days) after surgery. The primary clinical manifestations of AE-ILD are dyspnea, hypoxemia, rapid progression to respiratory failure, extensive ground-glass shadows in bilateral lungs on chest imaging, and acute interstitial pneumonia with rapid exacerbation. Mechanical ventilation was required in 85.7% (6/7) of patients with AE-ILD. The mortality rate of AE-ILD is 57.1% (4/7). The treatment and outcome of postoperative AE-ILD were shown in Table 3.

**Table 3:** Clinical characteristics and outcome of patients with postoperative AE-ILD

Gender /Age	Smoking index^a^/Quitting time	Comorbidity	PFT FEV_1_/MVV/DLCO (% pred)	Type and history of ILD	Image feature and range of ILD	Pathology and TNM stage	Surgery	Operation time (min)	Intraoperative blood loss (ml)	Onset of AE-ILD	Treatment	Outcome
M/74	30/180mo	None	80/84/53	IPF/3mo	UIP/whole lung field	SCLC/III-a	L + SLND/VATS	105	350	POD 2	Glucocorticoid /IMV	Died, POD 15
M/73	50/36mo	COPD, CHD	63.6/48.4/57.4	IPF/36mo	UIP/whole lung field	SCC/Ia2	W + SLND/VATS	75	30	POD 52	NIV	Improve
M/76	75/0.5mo	COPD, CHD, AF, renal carcinoma	92.8/93.4/64.9	IPF/36mo	UIP/bilateral subpleural area	SCLC/Ia1	W + SLND /VATS	60	20	POD 5	Glucocorticoid	Improve
M/60	150/1mo	CHD, DM	69/86/67	IPF/240mo	UIP/bilateral subpleural area	SCLC/IIb	L + SLND /VATS	160	200	POD 3	Glucocorticoid /NIV	Improve
M/74	50/1mo	HTN, SS, glomerular nephritis	88/134/66	CTD-ILD/48mo	Non-UIP/bilateral subpleural area	SCLC/IIIa	L + SLND /VATS	230	600	POD 3	Glucocorticoid /IMV	Died, POD 38
F/73	non-smoker	HTN, SS	60/92/64	CTD-ILD/48mo	Non-UIP/bilateral subpleural area	IP	L/VATS	160	600	POD 3	Glucocorticoid /NIV	Died, POD 13
M/70	non-smoker	CHD, DM	107.4/89/61.6	IPF/unknown	UIP/bilateral subpleural area	IP	S + W/VATS	100	50	POD 30	Glucocorticoid /NIV	Died, POD 60

Note: a: package-years

Abbreviation: AE-ILD: acute exacerbation of interstitial lung disease; AF: atrial fibrillation; CHD: coronary heart disease; COPD: chronic obstructive pulmonary disease; CTD-ILD: Connective tissue diseases related interstitial lung disease; DLCO: diffusing capacity of carbon monoxide; DM, diabetes mellitus; F: female; FEV1: forced expiratory volume in 1 s; HTN, hypertension; ILD: interstitial lung disease; IMV: invasive mechanical ventilation; IP: Inflammatory pseudotumor; IPF: idiopathic pulmonary fibrosis; L: lobectomy; M: male; min: minute; ml: milliliter; mo: month; MVV: maximal voluntary ventilation; NIV: Non-invasive ventilation; PFT: pulmonary function test; POD: postoperative day; S: segmentectomy; SCC: Squamous cell carcinoma; SCLC: Small cell carcinoma; SLND: systematic lymph node dissection; SS: Sjogren Syndrome; UIP: usual interstitial pneumonitis; VATS: video-assisted thoracoscopic surgery; W: wedge resection;

### Long-term complications and outcomes of ILD

A total of 12.8% (10/78) of patients experienced ILD exacerbation or other complications from 4 to 24 months postoperatively, including 4 cases of IPF, 3 cases of iNSIP, 2 cases of CTD-ILD, and 1 case of desquamative interstitial pneumonia. Three patients eventually died, one from AE-ILD (postoperative 6 months), one from ILD exacerbation and systemic multiple metastases (postoperative 6 months), and one from soft tissue infection of abdominal wall hernia (postoperative 4 months). The other 7 patients improved and were discharged after anti-infection, glucocorticoid and immunosuppressant, including 4 cases of ILD exacerbation combined with pulmonary infection (postoperative 5, 12, 16, 24 months, respectively), 1 case of ILD exacerbation combined with skin soft tissue infection (postoperative 4 months), 2 cases of ILD exacerbation (postoperative 4, 6 months, respectively) and 1 case of pulmonary infection (postoperative 4 months). The risk of complications occurring within or beyond postoperative 90 days in patients with long-term use of glucocorticoids and immunosuppressants was 59.3% (16/27), which was significantly higher than the risk (25.5%, 13/51) in patients without glucocorticoids and immunosuppressants, and the difference reached statistical significance (*p* < 0.05).

### Analyses of risk factors for postoperative 90-day complications

Based on the clinical data we collected and the high-risk factors reported in relevant studies through literature review, we screened high-risk factors that may affect perioperative complications. Finally, factors that reached statistical differences in the univariate logistic regression were included in the multivariate logistic regression. Univariate logistic regression analysis showed that comorbidity, age-adjusted Charlson Comorbidity Index, extent of resection, systemic lymph node dissection, operation time, intraoperative blood loss, and pathology of pulmonary lesion were identified as possible risk factors. In multivariate logistic regression analysis, age-adjusted Charlson Comorbidity Index and intraoperative blood loss were identified as independent high risk factors of postoperative 90-day complications. The results of univariate and multivariate logistic regression analyses were shown in Tables 4 and 5, respectively.

**Table 4:** Univariate logistic regression analysis of risk factors for postoperative 90-day complications

Variables	Number (%)	OR	95% CI	*P* value
**Age (> 65)**	45 (57.7)	2.8	0.899–8.717	0.076
**Gender (male)**	64 (84.2)	1.326	0.330–5.335	0.691
**Smoking history**				
Non-smoker	34 (43.6)	1.000	-	
Ex-smoker	20 (25.6)	1.538	0.405–5.842	0.527
Current smoker	24 (30.8)	3.333	0.824–13.482	0.091
Smoking index (package-years)		1.006	0.992–1.020	0.406
**Comorbidity**	58 (74.4)	9.256	1.151–74.412	**0.036**
**Age-adjusted Charlson Comorbidity Index**				
Score ≤ 3	44 (56.4)	1.000	-	**0.002**
Score > 4	34 (43.6)	6.158	1.948–19.470	
**PFT**				
FEV_1_ (% pred) < 70%	34 (43.6)	0.821	0.292–2.306	0.708
FVC (% pred) < 70%	24 (30.8)	0.684	0.216–2.163	0.518
FEV_1_/FVC (% pred) < 70%	21 (26.9)	0.875	0.273–2.804	0.822
MVV (% pred) < 70%	36 (46.2)	0.714	0.254–2.005	0.523
DLCO (% pred) < 60%	31 (39.7)	1.339	0.479–3.744	0.578
**Preoperative prophylaxis for ILD**	17 (21.8)	1.278	0.387–4.221	1.278
**History of AE-ILD**	14 (17.9)	1.815	0.527–6.251	0.345
**Surgical approach**				
VATS	68 (87.2)	1.000	-	
Thoracotomy	10 (12.8)	1.286	0.299–5.534	0.736
**Extent of resection**				
Sublobectomy	49 (62.8)	1.000	-	-
Lobectomy or more	29 (37.2)	3.618	1.256–10.424	**0.017**
**Systematic lymph node dissection**	50 (64.1)	4.293	1.132–16.278	**0.032**
**Operation time (min)**		1.010	1.002–1.018	**0.011**
**Intraoperative blood loss (ml)**		1.005	1.001–1.008	**0.008**
**Intraoperative accident or blood transfusion**	12 (15.4)	0.412	0.114–1.487	0.176
**Pathology of pulmonary lesion**				
Benign	28 (35.9)	1.000	-	
Malignant	50 (64.1)	4.293	1.132–16.278	**0.032**

Abbreviation: AE-ILD: acute exacerbation of ILD; CI: confidential interval; DLCO: diffusing capacity of carbon monoxide; FEV1: forced expiratory volume in 1 s; FVC: forced vital capacity; min: minute; MVV: maximal voluntary ventilation; ml: milliliter; OR: Odds ratio; PFT: pulmonary function test;

**Table 5:** Multivariate logistic regression analysis of risk factors for postoperative 90-day complications

Variables	OR	95% CI	*P* value
Age-adjusted Charlson Comorbidity Index (score > 4 vs. Score ≤ 3)	5.264	1.584–17.496	**0.007**
Intraoperative blood loss (ml)	1.004	1.001–1.008	**0.025**

Note: Unconditional multivariable logistic regression by backward selection was performed for age-adjusted Charlson Comorbidity Index, extent of resection, systemic lymph node dissection, operation time, intraoperative blood loss, and pathology of pulmonary lesion as initial variables. Variable selection was based on maximum likelihood estimation, with Inclusion α = 0.05 and Exclusion α = 0.10, respectively. Finally, the variables that reached the statistical difference were age-adjusted Charlson Comorbidity Index and intraoperative blood loss

Abbreviation: CI: confidential interval; ml: milliliter; OR: Odds ratio;

## Discussion

ILD patients tend to deteriorate after invasive procedure and pulmonary surgery, and several studies have reported a significantly higher risk of severe pulmonary complications after pulmonary resection in patients with idiopathic ILD [7, 8, 16–18]. However, there are very few reports on risk factors for postoperative complications in other types of ILD (e.g., iNSIP, CTD-ILD, etc.). Our study showed that the risk of postoperative 90-day complications was significantly higher in patients with IPF and CTD-ILD than in patients with iNSIP (35%, 27% vs. 13%). In univariate analysis, we found a strong relationship between the extent of pulmonary resection and postoperative 90-day complications, with significantly higher rates of overall complications (41.4% vs. 16.3%, *p* = 0.029) and pulmonary complications (27.6% vs. 8.2%, *p* = 0.048) after lobectomy and bilobectomy than sublobar resection, and no increased risk of complications with thoracotomy compared to VATS (30% vs. 25%, *p* = 1.000), consistent with the results reported by Sato et al [7]. However, the extent of pulmonary resection did not show a significant correlation with postoperative complications in the multivariate analysis, probably due to the influence of confounding factors such as operative time and intraoperative blood loss. Fibla et al. found open surgery to be an independent risk factor for postoperative 90-day mortality after surgical biopsy in patients with ILD [18–20]. Therefore, surgery for ILD patients should be as minimally invasive as possible.

In order to minimize postoperative risk, careful assessment of medical history and comorbidity are particularly important for ILD patients scheduled for surgery. Before performing invasive operations, emphasis should be placed on asking medical histories such as autoimmune disease, occupational and environmental exposure, along with pulmonary imaging features and other adjuvant examinations to comprehensively determine the type and severity of ILD. In this study, the risk of postoperative complications in patients with age-adjusted Charlson Comorbidity score greater than 4 was significantly higher (OR: 6.158, 95% CI: 1.948–19.470, *p* = 0.002), suggesting that age and comorbidities were strongly related to postoperative outcomes. Therefore, it is particularly important to fully evaluate the comorbidities and systemic organ functions (especially the pulmonary ventilation function and diffusion function) before surgery. Sudden reduction and withdrawal of therapeutic drugs may be a contributing factor to the exacerbation of ILD. The use of anti-fibrosis drugs, glucocorticoids, immunosuppressants and other drugs should be fully evaluated before surgery, so as to stabilize the condition of ILD and autoimmune diseases, avoid exacerbation of ILD and improve perioperative safety.

In this study, preoperative reduced pulmonary function was not found to increase postoperative complications. Of the 20 patients who developed overall complications, 30% (6/20) had moderate-to-severe ventilation dysfunction and 45% (9/20) had moderate-to-severe diffusion dysfunction; of the 12 patients who developed pulmonary complications, 42% (5/12) had moderate-to-severe ventilation dysfunction and 33% (4/12) had moderate-to-severe diffusion dysfunction. This suggests that ILD patients with normal or mildly decreased pulmonary function still have a high probability of developing complications after pulmonary resection. Sato et al. reported that preoperative vital capacity less than 80% of the predicted value was strongly associated with the risk of postoperative AE-ILD [21]. Ueno et al. reported that preoperative diffusing capacity of carbon monoxide less than 55% of the predicted value was associated with postoperative risk [22]. Therefore, reduced pulmonary function is an important risk factor for pulmonary resection in ILD patients.

Chest HRCT is the preferred method for screening and evaluation of ILD patients. The imaging features of ILD are ground glass opacities, grids, or honeycomb shadows in the lungs, sometimes with lobular septal thickening or distended bronchial dilatation, usually in the subpleural area, and in severe cases may be diffusely distributed in bilateral lungs. In our group, there were 61 (78.2%) and 17 (21.8%) cases of ILD distributed along the subpleural area and diffuse distribution in bilateral lungs, respectively. The incidence of overall complications (26.2% vs. 23.5%, *p* = 1.000) and pulmonary complications (14.8% vs. 17.6%, *p* = 1.000) was similar in the two groups. Although there is no statistical difference in our study, some studies have reported that the risk of postoperative complications (especially AE-ILD) is significantly increased in patients with usual interstitial pneumonia (UIP) type and extensive pulmonary involvement on HRCT [21, 23]. Therefore, preoperative HRCT is important to assess the extent and severity of ILD and helps to predict the risk of postoperative complications. Particular attention should be paid to pulmonary complications (especially AE-ILD) after surgery, and even if the postoperative recovery is successful, patients should be followed up for at least 3–6 months after discharge to prevent re-exacerbation of ILD.

AE-ILD is one of the most serious complications in the perioperative period in patients with ILD. Bronchoscopy, percutaneous lung puncture, pulmonary resection, chest radiotherapy, and anti-tumor drugs may induce AE-ILD, and the incidence of AE-ILD after pulmonary resection is as high as 11–40%, with a mortality rate of 30–100% [8]. Postoperative AE-ILD mostly occurs on postoperative days 2 to 10, and the incidence of involving ipsilateral lung, contralateral lung and bilateral lung was 14%, 25% and 61%, respectively [8]. Major pulmonary surgery, male sex, history of exacerbation, UIP appearance on chest CT, preoperative steroid use, serum sialylated carbohydrate antigen KL-6 levels greater than 1000 U/ml, and reduced percent predicted vital capacity are independent high-risk factors of postoperative AE-ILD [7, 21]. Therefore, respiratory symptoms and chest imaging changes should be closely monitored after pulmonary resection, especially the presence of pulmonary complications and AE-ILD.

Notably, one of the difficulties in the diagnosis and treatment of postoperative AE-ILD is that it is difficult to detect early. Possible reasons include: reduced pulmonary volume after pulmonary resection, airway obstruction, atelectasis, pleural effusion, increased incidence of pulmonary infection, pain at the surgical site, and circulatory volume overload, etc. These factors can easily lead to severe cough, wheezing, dyspnea, and hypoxemia, and their clinical manifestations and chest imaging changes are very similar to those of AE-ILD. Therefore, it is difficult to identify AE-ILD in the early stage of its onset, and the longtime of respiratory pathogenic results also leads to the delay of diagnosis, which bring difficulties to the treatment decision. If early diagnosis and timely intervention are not made, patients usually rapidly aggravate to respiratory failure and require mechanical ventilation. If rapidly progressive dyspnea occurs shortly after surgery, AE-ILD should be promptly identified, and HRCT and pathogenic examination should be performed as early as possible to avoid delay in its diagnosis and treatment.

AE-ILD is usually treated with high-dose glucocorticoid therapy. If glucocorticoids are ineffective, immunosuppressants (e.g., cyclophosphamide, tacrolimus, and cyclosporine A, etc.) can also be added. Early supportive care and mechanical ventilation should also be applied to patients. However, there is a lack of sufficient evidence and consensus for the above treatments [14]. For the use of glucocorticosteroids, we have the following experiences: (1). The time of medication should be early. Once AE-ILD is suspected, glucocorticosteroids should be used immediately. (2). The course of treatment should be sufficient. The use of glucocorticoids can be stopped only when the patient’s symptoms are completely improved and complete remission of interstitial pneumonia has been confirmed by chest CT. (3). The dose of glucocorticoids should be slowly reduced. Intravenous glucocorticosteroids should be gradually replaced by oral glucocorticosteroids and slowly reduced to discontinuation under stable conditions. (4). when the disease remains unstable, too rapid reduction or discontinuation of the glucocorticosteroids may easily lead to a re-exacerbation of the interstitial pneumonia. At this point, even if the dose of glucocorticoid is restored, the effect is usually not satisfactory. In our opinion, early, adequate doses and sufficient courses of glucocorticoids help to improve AE-ILD, and re-exacerbation should be avoided during treatment. New antifibrotic drugs (e.g., pirfenidone and nintedanib) contribute to slow the progression of IPF and reduce the risk of AE-IPF, and perioperative prophylactic use of pirfenidone may reduce the incidence of AE-IPF after pulmonary resection [24–26].

Considering the high risk of surgery in ILD patient, the timing and method of pulmonary resection should be carefully planned in cases with a long history of ILD and extensive pulmonary involvement. Minimally invasive surgery is preferred in patients with elderly age, unstable or severe ILD, significantly reduced pulmonary function, and severe comorbidities. The coexistence of ILD is an independent and unfavorable prognostic factor for lung cancer patients [10, 27]. Sato et al. reported that for patients with pathological TNM stage Ia, the prognosis after wedge resection was significantly worse than that after segmentectomy and lobectomy, and the 5-year overall survival was 29.2%, 60.0%, and 68.6%, respectively [9]. However, Tsutani et al. reported no significant difference in overall survival was observed between lobectomy and sublobar resection [28]. When planning surgical strategy for patients with ILD combined with lung cancer, a balance between radical resection and organ function preservation should be maintained, and the extent of pulmonary resection should be carefully selected. For low-risk patients with mild ILD and good cardiopulmonary function, radical lobectomy with systemic lymph node dissection should be preferred, but for high-risk patients with severe ILD and significantly reduced cardiopulmonary function, sublobar resection (segmentectomy is preferable to wedge resection) is more suitable.

### Study limitations

This study was a single-center, retrospective study with inevitable bias. The relatively small sample size of this study resulted in a small number of postoperative complications and AE-ILD events, which may lead to inadequate efficacy of statistical analysis. Due to the long-time span of the study enrollment and the limitations of the conditions at that time, surgical modalities and pharmacological treatments were presented in a variety of ways. Some patients with AE-ILD had difficulty in obtaining timely diagnosis, resulting in inconsistent perioperative outcomes and prognosis.

## Conclusions

Patients with ILD have a significantly higher risk of 90-day complications and mortality after pulmonary resection, especially pulmonary complications and AE-ILD. AE-ILD often starts 2 to 3 days postoperatively, and a high proportion of patients require mechanical ventilation with mortality rates exceeding 50%. After postoperative 90 days, the risk of deterioration of pulmonary status remains high, including exacerbation of ILD and complications associated with long-term use of glucocorticoids and immunosuppressant. Age, comorbidity and intraoperative blood loss are high risk factors for postoperative 90-day complications. Therefore, both the short-term risks and the long-term risks following pulmonary resection in patients with ILD should receive adequate attention from thoracic surgeons during the perioperative and follow-up periods.

## Data Availability

All data generated or analyzed during this study are included in this article.

## Abbreviations

ILD: interstitial lung disease
IPF: idiopathic pulmonary fibrosis
iNSIP: idiopathic nonspecific interstitial pneumonia
DIP: desquamative interstitial pneumonia
CTD-ILD: connective tissue diseases related interstitial lung disease
LC: lung cancer
AE-ILD: acute exacerbation of interstitial lung disease
HRCT: high-resolution computed tomography
TNM: Tumor, Node, Metastasis
MRI: magnetic resonance image
VATS: video-assisted thoracoscopic surgery
cm: centimeter
OR: Odds ratios
CI: confidence intervals
